# A novel esterase from a marine mud metagenomic library for biocatalytic synthesis of short-chain flavor esters

**DOI:** 10.1186/s12934-016-0435-5

**Published:** 2016-02-18

**Authors:** Wenyuan Gao, Kai Wu, Lifeng Chen, Haiyang Fan, Zhiqiang Zhao, Bei Gao, Hualei Wang, Dongzhi Wei

**Affiliations:** State Key Laboratory of Bioreactor Engineering, New World Institute of Biotechnology, East China University of Science and Technology, Shanghai, 200237 People’s Republic of China

**Keywords:** Metagenomic library, Functional screening, Esterase, Transesterification, Short-chain flavor esters, High substrate loading

## Abstract

**Background:**

Marine mud is an abundant and largely unexplored source of enzymes with unique properties that may be useful for industrial and biotechnological purposes. However, since most microbes cannot be cultured in the laboratory, a cultivation-independent metagenomic approach would be advantageous for the identification of novel enzymes. Therefore, with the objective of screening novel lipolytic enzymes, a metagenomic library was constructed using the total genomic DNA extracted from marine mud.

**Results:**

Based on functional heterologous expression, 34 clones that showed lipolytic activity were isolated. The five clones with the largest halos were identified, and the corresponding genes were successfully overexpressed in *Escherichia coli*. Molecular analysis revealed that these encoded proteins showed 48–79 % similarity with other proteins in the GenBank database. Multiple sequence alignment and phylogenetic tree analysis classified these five protein sequences as new members of known families of bacterial lipolytic enzymes. Among them, EST4, which has 316 amino acids with a predicted molecular weight of 33.8 kDa, was further studied in detail due to its strong hydrolytic activity. Characterization of EST4 indicated that it is an alkaline esterase that exhibits highest hydrolytic activity towards *p*-nitrophenyl butyrate (specific activity: 1389 U mg^−1^) at 45 °C and pH 8.0. The half-life of EST4 is 55 and 46 h at 40 and 45 °C, respectively, indicating a relatively high thermostability. EST4 also showed remarkable stability in organic solvents, retaining 90 % of its initial activity when incubated for 12 h in the presence of hydrophobic alkanes. Furthermore, EST4 was used as an efficient whole-cell biocatalyst for the synthesis of short-chain flavor esters, showing high conversion rate and good tolerance for high substrate concentrations (up to 3.0 M). These results demonstrate a promising potential for industrial scaling-up to produce short-chain flavor esters at high substrate concentrations in non-aqueous media.

**Conclusions:**

This manuscript reports unprecedented alcohol tolerance and conversion of an esterase biocatalyst identified from a marine mud metagenomic library. The high organic solvent tolerance and thermostability of EST4 suggest that it has great potential as a biocatalyst.

**Electronic supplementary material:**

The online version of this article (doi:10.1186/s12934-016-0435-5) contains supplementary material, which is available to authorized users.

## Background

Lipolytic enzymes, including esterases and lipases, belong to the general class of carboxylic ester hydrolases (EC 3.1.1) that catalyze both the hydrolysis and formation of ester bonds. While carboxylesterases (EC 3.1.1.1) hydrolyze water-soluble or emulsified esters with short-chain carboxylic acids (˂10 carbon atoms), lipases (EC 3.1.1.3) prefer long-chain fatty acids (≥10 carbon atoms), even though the characteristic α/β hydrolase fold is found in the three-dimensional structure of both the enzymes [1, 2]. These biocatalysts generally do not require cofactors and are remarkably stable in organic solvents. In addition, the broad substrate specificity, high stereoselectivity, and high positional selectivity of these biocatalysts make them useful for the production of enantiopure secondary alcohols and the resolution of primary alcohols and carboxylic acids [3–5].

There is an increasing demand for novel biocatalysts in modern industry, which has prompted the development of novel approaches to isolate biocatalyst-encoding genes. However, the identification of novel biocatalysts from microorganisms is limited by the fact that only 1 % of microorganisms can be cultured using conventional laboratory methods [6]. Fortunately, metagenomics, which is a cultivation-independent method, can be used to avoid this inherent loss of diversity and is regarded as one of the most powerful approaches to investigate the potential of particular microorganisms without the need for culturing [7]. Indeed, the metagenomic approach was useful in retrieving various enzymes of biotechnological importance, such as amidase, amylase, protease, and alcohol oxidoreductase [7]. In addition, numerous lipolytic enzymes have been successfully identified from the metagenomic libraries of different environmental samples, such as deep-sea sediment [8], hot spring sediment [9], intertidal flat sediment [10], forest soil [11], activated sludge [12–14], compost [15], and pond water [16]. Therefore, there is great interest in further metagenomic-based searches for novel enzymes from different sources and with greater industrial applicability.

Though metagenomic technology is efficient to discover novel enzymes, there are still some limitations. Insufficient purification of soil DNA might lead to interference with cloning because of the coextracted humic acids, while higher purification levels may incur losses of genetic information. The expression system of heterologous genes is hampered by inefficient transcription of target genes as well as improper assembly of the corresponding enzymes. Furthermore, it is difficult to establish the high-throughput screening for identify millions of positive clones in a metagenomic library in a short time, because it depends on the nature of target protein [17].

Short-chain fatty acid esters are commonly used in the food, beverage, cosmetic, and pharmaceutical industries as flavorings or fragrances due to their typical fruity smells and high volatilities [18]. Traditionally, most flavor compounds are obtained by chemical synthesis or extraction from natural sources [19, 20]. Whereas natural flavor esters extracted from plant materials are often too scarce or expensive for industrial use. On the other hand, chemical synthesis often involves environmentally harmful production processes and lacks substrate selectivity, which may produce racemic mixtures with undesired side products that reduce synthesis efficiency and increase downstream costs [21]. In addition, the products cannot legally be labeled as natural. The disadvantages of these methods and the high demand for natural flavor esters have led industries to seek new strategies for the production of flavor compounds. Esterification and transesterification by lipolytic enzymes are among the most effective alternatives to the chemical synthesis of short-chain flavor esters. Nevertheless, lower substrate concentrations and conversion rates have constrained the commercial scale-up of enzyme-mediated catalysis.

In this study, we constructed a fosmid metagenomic library from marine mud for large-scale functional screening of lipolytic genes. Five clones with lipolytic activity were detected, and a novel esterase (EST4) with the highest activity was selected from the target clones for further characterization. EST4 displayed excellent catalytic activity for the synthesis of flavor esters in non-aqueous media with high substrate concentrations.

## Results and discussion

### Construction and characterization of a marine mud metagenomic library

Fosmids are good vectors for constructing metagenomic libraries due to their high cloning efficiency, improved stability in *Escherichia coli*, and optimal (40 kb) insert size [22]. A total yield of approximately 1.5 μg of 40 kb high-quality DNA was obtained, as described in the Methods (Additional file 1: Figure S1). The marine mud metagenomic library revealed more than 40,000 fosmid clones and represented about 1.6 Gb of the microbial community DNA. Given an average prokaryotic genome of approximately 4 Mb, the library reached a theoretical size of over 400 genomes. An analysis of the insert fragments by digestion of 10 randomly selected clones with *Not*I indicated that 90 % of the clones contained different inserts with an average size of 40 kb (Additional file 2: Figure S2). This restriction analysis suggests that the metagenomic library is of high quality and diversity.

### Functional screening and identification of lipolytic clones

**Fig. 1:**
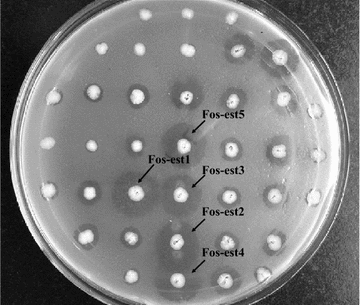
Hydrolysis halos formed by different clones isolated from marine mud metagenomic library. Activity was observed on 0.5 % (v/v) tributyrin containing LB agar after 48 h of incubation at 37 °C. Five clones showed the largest hydrolysis halos and were chosen for further study

Functional screening of the metagenomic library for lipolytic activity was based on the hydrolytic ability of the clones growing on tributyrin-containing LB chloramphenicol plates. All positive fosmids were extracted from the original clones and then retransformed into *E. coli.* The new transformants were plated on the same selective medium. Finally, the re-transformants were characterized by the presence of hydrolysis halos. As a result, thirty-four clones showed hydrolysis halos after incubation for 48 h at 37 °C (Fig. 1). The halo size of different clones for tributyrin hydrolysis varied from 2 to 14 mm, indicating variable expression or substrate preference of the lipolytic enzymes produced by the clones. The duplicate clones were removed after a restriction enzyme treatment with *Bam*HI (Additional file 3: Figure S3). The five clones, which showed the highest hydrolytic activity toward tributyrin, were selected for further characterization. Based on the hydrolysis activity of *p*-nitrophenyl (*p*NP) esters with different acyl chain lengths in subsequent experiments (data not shown), all the five enzymes preferred to hydrolyze short acyl chain substrates (C < 10), and thus were named as Fos-est1, Fos-est2, Fos-est3, Fos-est4, and Fos-est5.

### Sub-cloning and sequence analysis

In order to identify the corresponding genes in the fosmids that encode the enzymes showing lipolytic activity, the inserts were further refined through sub-cloning experiments. The inserted DNA of each of the five lipolytic clones was fragmented to a size of 2.5–4.5 kb and cloned into pBluescript II SK(+), producing a sub-clone library of >10^3^ clones. The sub-clones that expressed extracellular lipolytic activity were sequenced. Five open reading frames (ORFs) encoding the potential lipolytic genes were identified based on ORF finder analysis and BlastP alignments, and were designated *est1*, *est2*, *est3*, *est4*, and *est5*. None of putative gene products was identical to a known or putative protein, as revealed by BlastP analysis based on the information in the GenBank database. The products exhibited low identity (48–79 %) with the proteins from *Cupriavidus metallidurans* [GenBank: WP_024569139]*, Novosphingobium nitrogenifigens* [GenBank:WP_008066710]*, Actinobacterium acAcidi* [GenBank: KGA09150 and KGA09147], and unclassified bacteria [GenBank: AAZ48934] (Table 1).

**Table 1:** Properties of the lipolytic proteins from marine mud metagenomic library and similar proteins in GenBank

ORF	G + C (%)	Size (aa^a^)	Closest protein and accession no.	Microorganism	% Identity/similarity	Score^b^	E value^c^
*est1*	54.6	301	Hypothetical protein GM46_7020 (KGA09150)	*Actinobacterium acAcidi*	79/86	489	2e − 171
*est2*	65.4	316	Lipase (WP_008066710)	*Novosphingobium nitrogenifigens*	62/75	398	7e − 135
*est3*	58.3	442	Putative beta-lactamase class C (AAZ48934)	Uncultured bacterium WWRS-2005	77/86	642	0.0
*est4*	52.9	316	Alpha/beta hydrolase (WP_024569139)	*Cupriavidus metallidurans*	48/65	304	3e − 98
*est5*	51.8	302	Hypothetical protein GM46_7005 (KGA09147)	*Actinobacterium acAcidi*	76/88	486	4e − 170

^a^Length of predicted ORF in amino acids

^b^Bit score of alignment using BLAST

^c^The value is a parameter that describes the number of hits one can “expect” to see by chance when searching a database of a particular size

### Phylogenetic relationships of the novel lipolytic enzymes

**Fig. 2:**
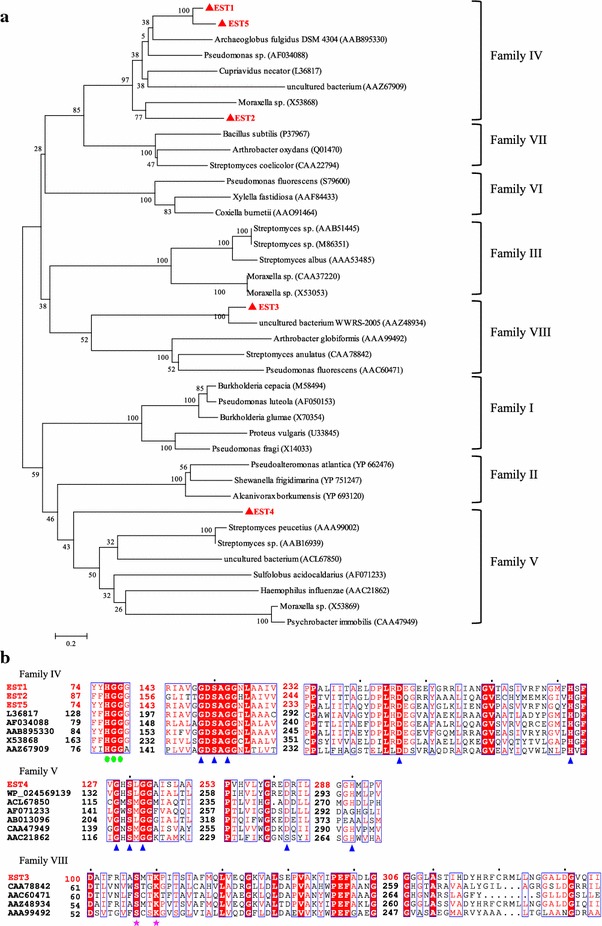
Bioinformatic analysis of lipolytic enzymes. **a** Phylogenetic analysis of lipolytic enzymes and closely related proteins. Phylogenetic analysis was performed using Clustal X and MEGA 6.0. The lipolytic enzymes found in this study are shown as red triangles. Only bootstrap values higher than 50 % are shown. The *scale bar* represents 0.2 changes per amino acid. **b** Multiple sequence alignment of conserved regions of lipolytic enzymes belonging to families IV, V, and VIII. Sequence alignment was performed using Clustal X and ESPript 3.0. Conserved sequences are indicated with *boxes*, and similar sequences are indicated using a *colored background*. The catalytic triads (*blue triangles*) and the typical motifs of family IV (*green circles*) and family VIII (*pink stars*) are identical

For the phylogenetic analysis, bacterial lipolytic enzyme references [1] representing eight different bacterial families were selected. All the deduced amino acid sequences of the five lipolytic genes differed from each other, but showed similarities to various types of lipolytic enzymes or hydrolases in different families (Fig. 2). As shown in Fig. 2, the five enzymes can be grouped into three distinct lipase/esterase reference families (IV, V, and VIII). EST4 relates to family V in the phylogenetic tree and contains a catalytic triad that is typical of proteins with an α/β hydrolase fold. The phylogenetic analysis indicated that EST3 belongs to family VIII, which shows a striking similarity to several class C β-lactamases. In fact, the S-M-T-K sequence found in *est3* corresponds to the S-x-x-K motif, which is conserved both in class C β-lactamases [23] and family VIII carboxylesterases [1]. Furthermore, the G-x-S-x-G motif common to some lipolytic protein families and present in some members of the family VIII esterase is absent from EST3 and closely related proteins. EST1, EST2, and EST5 belong to family IV, which displays a striking amino acid sequence similarity to mammalian hormone-sensitive lipase (HSL). Multiple-sequence alignment revealed that these three enzymes contain the typical H-G-G–G motif and the lipase-conserved catalytic triad Asp-His-Ser in the consensus pentapeptide G-x-S-x-G. These results suggest that these three enzymes are new members of the HSL family.

### Heterologous expression of lipolytic genes and purification of EST4

**Fig. 3:**
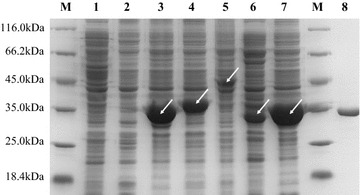
SDS-PAGE analysis of soluble lysates of lipolytic enzymes and the purified esterase EST4. *Lane* M, molecular weight protein marker (Thermo Scientific, Cat. No: 26610); *lane 1*, soluble lysates of *E. coli* Top10Fʹ/pLLP-OmpA, as negative control; *lane 2*, soluble lysates of *E. coli* BL21/pET28a, as negative control; *lane 3*, EST1; *lane 4*, EST2; *lane 5*, EST3; *lane 6*, EST4; *lane 7*, EST5; *lane 8*, purified EST4 (34 kDa). The *arrows* indicate expected proteins from recombinant *E. coli*

Each of the four lipolytic genes (*est1*, *est2*, *est3*, and *est5*) was amplified, cloned into the pET-28a (+) vector with a 6× His tag at the N-terminus, and transformed into *E. coli* BL21 (DE3) cells for expression. As the *est4* gene was expressed in inactive inclusion body in the pET-28a (+) vector, combinations of various vectors and hosts were tested and it was ultimately overexpressed in *E. coli* Top10F′/pLLP-OmpA with a C-terminal His_6_-tag. Detailed strategies for the soluble expression of EST4 are described in Additional file 4. All the encoded proteins were successfully overexpressed in an active form with the expected molecular weights (32–48 kDa) (Fig. 3). Most of lipolytic enzymes demonstrated high expression levels without extensive optimization of the cultivation and induction conditions, which indicates that these enzymes are inherently amenable to overexpression in *E. coli*. Among the five lipolytic enzymes, EST4 was studied in further detail owing to its strong hydrolytic activity toward tributyrin (Additional file 5: Figure S4). Based on the terminal 6× His affinity tag, the EST4 was purified to electrophoretic homogeneity through nickel affinity chromatography. The purified EST4 was separated as a single protein band of approximately 34 kDa by sodium dodecyl sulfate polyacrylamide gel electrophoresis (SDS-PAGE) (Fig. 3), consistent with the molecular weight of 33.8 kDa deduced from the *est4* amino acid sequence.

### Substrate specificity

**Fig. 4:**
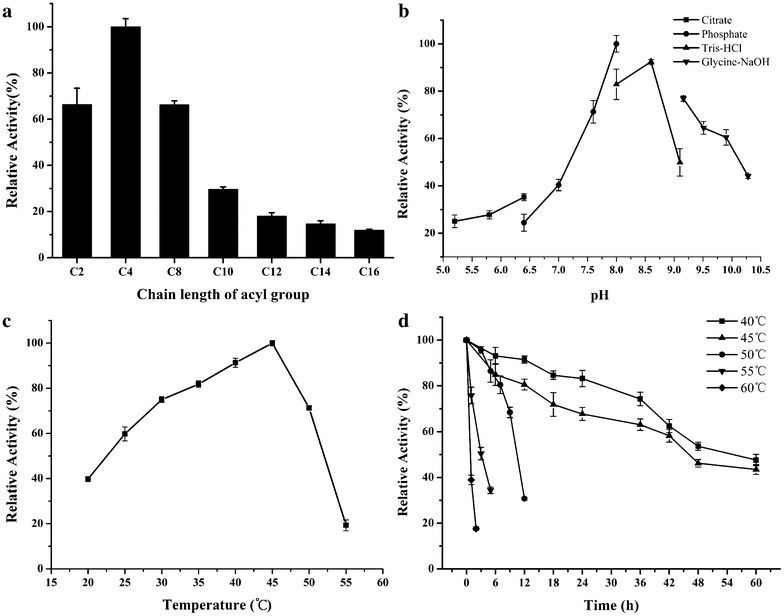
Characterization of EST4. **a** Substrate specificity of EST4 on various *p*-nitrophenyl esters. *p*-nitrophenyl esters of various lengths were assayed at 30 °C in 100 mM Tris–HCl buffer, pH 8.0. **b** Effect of pH on EST4 activity, measured at 30 °C for 5 min in 100 mM different buffers with varying pH values using *p*-NP butyrate as the substrate. The buffers used were citrate (*solid square*), phosphate (*solid circle*), Tris–HCl (*solid triangle*), and Glycine-NaOH (*solid inverted triangle*). **c** Effect of temperature on EST4 activity, measured at different temperatures for 5 min in 100 mM Tris–HCl buffer, pH 8.0. **d** Effect of temperature on EST4 stability. EST4 was incubated in 100 mM Tris–HCl buffer, pH 8.0, at 40, 45, 50, 55, or 60 °C for various durations, and residual activity was measured at 30 °C for 5 min. The maximal activity was defined as 100 % and the relative activity is shown as a percentage of maximal activity

Based on substrate preference, lipolytic enzymes are categorized as lipases that hydrolyze ester bonds of water-insoluble or emulsified lipid substrates which have long acyl chains (carbon chain length ≥10) or esterases that show highest activity toward water-soluble or emulsified esters with relatively short fatty acid chains (carbon chain length <10) [24]. In order to determine the substrate specificity of EST4, we tested its ability to hydrolyze *p*-nitrophenyl (*p*-NP) esters with various acyl chain lengths under standard assay conditions. EST4 was able to hydrolyze *p*-NP esters with acyl chains of up to 16 carbons (*p*-NP palmitate). EST4 displayed higher activity for short-chain fatty acids (C < 10) and the highest specific activity was 1389 U∙mg^−1^ with *p*-NP butyrate (C4; pH 8.0 and 45 °C). Lower levels of activity were observed with longer chain fatty acids (C ≥ 10) (Fig. 4a). These results indicate that EST4 is a “true” esterase that preferentially hydrolyzes short acyl chain substrates [1, 25].

### Effect of temperature and pH on enzyme activity and thermostability

The effects of pH and temperature on the enzymatic activity of EST4 were investigated using *p*-NP butyrate (C4) as a test substrate. The enzyme displayed a maximum activity at pH values between 7.6 and 8.6, and the optimal pH was 8.0. EST4 was rapidly inactivated at lower pH values (pH < 7.0). However, EST4 showed relatively high activity under alkaline conditions and retained approximately 76 % of the maximum activity at pH 9.16. Thus, we concluded that EST4 is an alkaline esterase (Fig. 4b). EST4 displayed activity over the broad range from 20 to 60 °C (Fig. 4c), with an optimal activity at 45 °C. The esterase activity increased linearly with temperature up to 45 °C, and then decreased at higher temperatures.

The thermostability of EST4 was determined by analyzing the residual activity at various time intervals after pre-incubation of the purified enzyme for up to 60 h at various temperatures (40–60 °C; Fig. 4d). EST4 exhibited good thermostability with residual activity of approximately 50 % after incubation at 40 or 45 °C for 55 or 46 h, respectively. At 50 °C, the half-life of EST4 was 10 h, whereas at 55 or 60 °C, the residual activity decreased sharply within 3 h. Based on the optimal activity temperature and thermostability of the purified enzyme, EST4 can be used efficiently within a broad temperature range and is suitable for biotechnological applications performed at high temperatures.

### Effect of detergents, metal ions, and organic solvents on EST4 activity

The activity of EST4 was measured in the presence of metal ions and ethylenediaminetetraacetic acid (EDTA; Table 2). These tests did not show any significant stimulation of the enzyme activity. The addition of 5 mM of metal ions decreased the esterase activity greatly by 20–70 % (with Mg^2+^ and Ca^2+^ being exceptions). The addition of 1 mM of Mg^2+^, Ca^2+^, Mn^2+^, Fe^2+^, and Cu^2+^ inhibited the esterase activity slightly by up to 10 %, whereas the addition of 1 mM of Ni^2+^, Fe^3+^, Co^2+^, and Zn^2+^ reduced the activity by more than 20 %. The chelating agent EDTA had no significant influence on EST4 activity. These findings indicate that EST4 activity does not require the presence of metal ions and that the structure of EST4 does not contain metal-binding sites, meaning that EST4 is not a metalloenzyme. Therefore, EST4 is similar to the metagenomic esterase EstMY [13] and esterase Est_p1 identified from a marine sediment metagenomic library [26].

**Table 2:** Effect of metal ions on EST4 activity

Ion	Relative activity (%)^a^	
	1 mM	5 mM
Control	100.0 ± 4.2	100.0 ± 0.8
EDTA	97.0 ± 3.6	95.2 ± 2.1
Mg^2+^	95.1 ± 2.9	92.2 ± 0.8
Ca^2+^	94.1 ± 2.9	98.0 ± 1.0
Mn^2+^	92.4 ± 4.7	83.8 ± 3.6
Fe^2+^	87.6 ± 3.3	57.4 ± 1.0
Cu^2+^	87.1 ± 5.3	66.9 ± 4.8
Ni^2+^	78.8 ± 2.5	67.1 ± 2.4
Fe^3+^	77.0 ± 3.0	57.1 ± 4.8
Co^2+^	74.5 ± 4.0	44.5 ± 5.8
Zn^2+^	63.6 ± 3.0	31.6 ± 3.5

^a^The activity toward *p*-nitrophenyl butyrate without any metal ions was taken as 100 %. All measurements were repeated three times

The addition of various detergents had varying effects on esterase activity (Table 3). A slight increase in esterase activity was observed upon the addition of 0.5 % Tween 60 (109 %) and Tween 80 (122 %) in comparison with the control, after 0.5 h pre-incubation at 30 °C. However, the addition of 0.5 % Tween 20, Tween 40, and Triton X-100 did not affect the lipolytic activity (98, 103, and 99 %, respectively), whereas ionic surfactants, such as sodium dodecyl sulfonate (SDS) and cetyltrimethylammonium bromide (CTAB), had a strong inhibitory effect on the esterase activity.

**Table 3:** Effect of detergents on EST4 activity

Detergent	Relative activity (%)^a^
Control	100.0 ± 4.2
Tween 20	97.62 ± 1.3
Tween 40	102.90 ± 2.8
Tween 60	108.71 ± 2.1
Tween 80	122.50 ± 1.4
Triton X-100	99.29 ± 5.3
CTAB	ND^b^
SDS	0.26 ± 0.6

^a^The activity toward *p*-nitrophenyl butyrate without detergents was set as 100 %. All measurements were repeated three times

^b^ *ND* not detectable

In order to study comprehensively the effect of organic solvents on EST4, various concentrations of different types of organic solvents were investigated (Table 4). EST4 activity decreased after 12 h of incubation with increasing concentrations of hydrophilic organic solvents (DMSO, DMF, methanol, and ethanol). Interestingly, the residual activity of EST4 noticeably increased in neat hydrophilic solvents, such as acetone, acetonitrile, and isopropanol, retaining 85.2, 89.0, and 97.0 % of the initial activity, respectively, after 12 h of incubation. Due to the stripping effects of hydrophilic organic solvents, which sequester bound water molecules from the enzyme, an increase of hydrophilic solvent content in the reaction medium results in reduction of enzyme activity. However, very high enzyme activities can be obtained in certain neat hydrophilic solvents, such as isopropanol and acetone, which are capable of forming multiple hydrogen bonds with enzyme molecules, thereby stabilizing the transition state of the reaction, and partially mimicking the effect of water [27, 28]. This profile is similar to that of the lipase from *Serratia marcescens* ECU1010 reported by Zhao et al. [29] and the SML lipase from *Stenotrophomonas maltophilia* CGMCC 4254 reported by Li et al. [30].

**Table 4:** Effects of organic solvents on EST4 activity

Organic solvents	log *P* ^a^	Residual activity (%)^b^ at concentration (%, v/v) of		
		20	50	100
Control	–	100.0 ± 2.1	100.0 ± 3.8	100.0 ± 0.5
DMSO	−1.3	82.8 ± 1.7	61.7 ± 8.6	0.6 ± 1.5
DMF	−1.0	92.1 ± 2.5	19.4 ± 4.2	5.8 ± 2.0
Methanol	−0.76	90.6 ± 9.4	23.2 ± 1.8	1.3 ± 3.2
Ethanol	−0.24	102.0 ± 7.7	0.5 ± 2.4	4.9 ± 2.1
Acetone	−0.23	53.1 ± 6.4	ND^c^	85.2 ± 1.3
Acetonitrile	−0.15	96.2 ± 2.7	7.8 ± 1.4	89.0 ± 4.3
Isopropanol	0.1	81.3 ± 7.9	ND	97.0 ± 0.9
Benzene	2.0	37.9 ± 3.3	76.1 ± 4.7	92.8 ± 4.3
Toluene	2.5	63.5 ± 1.6	88.1 ± 4.0	90.1 ± 0.5
Cyclohexane	3.2	80.1 ± 2.7	92.7 ± 5.9	99.7 ± 1.8
*n*-hexane	3.5	53.1 ± 4.6	95.3 ± 10.6	98.6 ± 0.5
*n*-heptane	4.0	66.1 ± 2.8	96.9 ± 5.6	97.6 ± 1.3
Isooctane	4.5	85.5 ± 5.4	90.0 ± 10.9	98.4 ± 1.0

^a^log *P* value is the partition coefficient of an organic solvent between water and *n*-octanol phases

^b^After incubating EST4 for 12 h in different organic solvents, the residual enzymatic activity was measured in 100 mM Tris–HCl buffer (pH 8.0) at 30 °C using *p*-NP butyrate as the test substrate. An enzyme sample incubated in buffer only was used as the measure of 100 % activity

^c^ *ND* not detectable

EST4 was apparently more stable in hydrophobic organic solvents than in hydrophilic organic solvents. The esterase was extremely stable in hydrophobic organic solvents (log *P* ≥ 2.0) at both 50 and 100 % (v/v) concentration, retaining approximately 90 % of its original activity after incubation for 12 h (Table 4). Generally, many synthetic reactions catalyzed by lipolytic enzymes are carried out in water-immiscible organic solvents with small amounts of water [31–33]. Therefore, the remarkable stability of EST4 makes it an attractive candidate for transesterification and ester synthesis reactions that involve high log *P* solvents such as *n*-hexane [34]. Although some previous studies describe a noticeable tolerance of esterase for organic solvents [35], there are no reports of an organic solvent-tolerant and thermostable esterase from a marine mud metagenomic library.

### Potential application of EST4 in the synthesis of short-chain flavor esters

Esterases have often been used to synthesize short-chain flavor esters (e.g., cinnamyl acetate, citronellyl acetate, geranyl acetate, and isoamyl acetate) through transesterification in non-aqueous systems. Unfortunately, the biosynthesis of alcohol esters is often limited by low substrate concentrations, as high amounts of water-soluble alcohols denature the enzyme by interfering with the enzyme-bound water layer [36].

**Scheme1:**
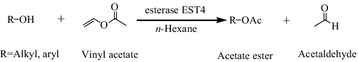
Esterase catalyzed transesterification reaction of flavor alcohol with vinyl acetate

**Fig. 5:**
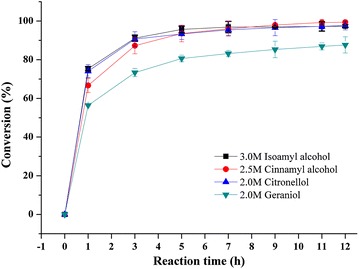
Time-course of the synthesis of various short-chain flavor esters at different substrate concentrations by EST4. Solvent *n*-hexane, reaction temperature 40 °C, alcohol/acyl donor molar ratio 1:2, whole-cell biocatalyst 10 g L^−1^, shaking speed 200 rpm

In order to explore the potential application of EST4 to the synthesis of various short-chain flavor esters, the lyophilized *E. coli* as a whole-cell biocatalyst was used for the synthesis of cinnamyl acetate, citronellyl acetate, geranyl acetate, and isoamyl acetate at high substrate concentrations in non-aqueous systems (Scheme 1). Since esterase can simultaneously catalyze both hydrolysis and transesterification reactions, excess water would promote the hydrolysis of product. In order to control the water content, we used dry cell powder of EST4 as whole-cell biocatalyst for the synthesis of the flavor esters through transesterification. The time courses of these reactions are depicted in Fig. 5. As it can be seen in Fig. 5, this biocatalyst can tolerate alcohol concentrations greater than 2.0 M to achieve up to 99 % conversion with isoamyl alcohol, cinnamyl alcohol, and citronellol, while geraniol had a comparatively low conversion (88 % in 12 h). It is notable that 382 g L^−1^ isoamyl acetate (98 % conversion of 3.0 M alcohol in 12 h) was produced, which is higher than the values reported in the literature for the transesterification reaction [37–39]. It should be emphasized that isoamyl alcohol is almost completely depleted for concentrations up to 3.0 M, demonstrating the excellent potential of esterase EST4. These results also agree with the excellent stability of EST4 in organic media.

From the applications viewpoint, a high substrate concentration is beneficial for enzymatic processes since it can improve the space–time yield and greatly reduce the cost of product isolation. In the present study, short-chain esters were efficiently synthesized by esterase EST4 using substrate concentrations that were significantly higher than those used in other reports [40–42]. Dhake et al. reported a cinnamyl acetate yield of 99 % in 24 h with an immobilized lipase from *Rhizopus oryzae* using a fairly low substrate concentration (0.33 M) [40]. For the production of geranyl acetate in solvent-free systems with an immobilized lipase from *Rhizopus oligosporus* NRRL 5905, a maximum molar conversion of 67 % was achieved with 100 mM substrate after 48 h at 30 °C [42]. To the best of our knowledge, this manuscript is the first report of an esterase biocatalyst with such high alcohol tolerance and high conversion rate, and further providing remarkable resistance to ester and alcohol denaturation during biosynthesis of various short-chain flavor esters. These results proved that EST4 is one of the most promising biocatalysts for the synthesis of short-chain flavor esters at high substrate concentrations and has great potential for large-scale commercial production of flavor esters.

## Conclusions

In summary, we identified a novel thermostable esterase, EST4, from a marine mud metagenomic library. EST4 is highly stable in organic solvents and it efficiently synthesized various short-chain flavor esters in non-aqueous media under high substrate concentrations (up to 3.0 M). The outstanding characteristics of EST4 make it a potential candidate for the industrial biosynthesis of relevant short-chain flavor esters under mild conditions. This study also broadens the diversity of lipolytic genes and demonstrates that the metagenomic approach is a useful technique for discovering novel enzymes with potential for industrial applications.

## Methods

### Chemicals and reagents

Phanta super-fidelity DNA polymerase (Vazyme, Nanjing, China) was used for DNA amplification. Alkaline phosphatase, calf intestinal (CIP) and restriction enzymes *Not*I and *Sau*3AI were purchased from New England BioLabs (Ipswich, MA, USA). Other restriction enzymes and T4 DNA ligase were supplied by MBI Fermentas (Baltimore, MD, USA). The *p*-nitrophenyl ester series, *p*-nitrophenol, citronellol, geraniol, cinnamyl alcohol, and isoamyl alcohol were purchased from Sigma-Aldrich (St. Louis, MO, USA). All other commercially available chemicals and solvents were of analytical or higher grade.

### Bacterial strains, plasmids, and growth conditions

The *E. coli* EPI300™-T1R and pCC1FOS fosmid vectors (CopyControl Fosmid Library Production Kit, Epicentre Biotechnologies, Madison, WI, USA) were used to construct the metagenomic library. *E. coli* DH5α and the pBluescript II SK(+) vector (Stratagene, Heidelberg, Germany) were used for the sub-cloning steps and sequencing. *E. coli* BL21 (DE3)/pET-28a (+) (Novagen, Madison, MI, USA) and *E. coli* Top10Fʹ/pLLP-OmpA (Seebio, Shanghai, China) were used as the recombinant protein expression system. *E. coli* cells were grown aerobically at 37 °C in Luria–Bertani (LB)-Miller medium supplemented with appropriate antibiotics.

### DNA extraction and purification from marine mud

The marine mud was obtained from the Yellow Sea, China. The marine mud’s metagenomic DNA was isolated using the Mo Bio Power Soil DNA isolation kit (MO BIO Laboratories, Inc., Carlsbad, CA, USA). In order to remove any co-extracted humic substances and other contaminants, the metagenomic DNA was further purified and concentrated through ethanol precipitation.

### Metagenomic library construction and screening

The metagenomic library was constructed using the CopyControl pCC1FOS Fosmid Library Production Kit (Epicentre Biotechnologies) following the manufacturer’s protocol. Transformants were grown on LB agar with 12.5 μg mL^−1^ chloramphenicol (Chl) at 37 °C for 20 h, washed with LB medium containing 10 % glycerol, and stored at −80 °C. To validate the library, 10 clones were randomly selected and fosmid DNAs were purified using the alkaline lysis protocol [43]. The positive controls contained Fosmid Control DNA (Epicentre Biotechnologies), and the negative controls contained *E. coli* host DNA without any fosmid. Digestion with the restriction enzyme *Not*I and further visualization in an agarose gel revealed that the average size of the cloned fragments was about 40 kb and that the DNA inserts were different.

For lipolytic activity screening, the metagenomic library pools were appropriately diluted with sterile water, incubated on LB agar containing 12.5 μg mL^−1^ Chl and 0.5 % (v/v) emulsified tributyrin at 37 °C for 2–3 days [44]. Clones with clear halos around individual colonies, which indicated hydrolysis of tributyrin, were chosen as positive clones. To confirm that the observed phenotype could be attributed to the metagenomic DNA insert, the fosmid DNAs were purified using an alkaline lysis protocol [43] and retransformed on the same type of indicator plate.

### Sub-cloning and sequence analysis of lipolytic genes

The pooled insert DNA fragments from the fosmids of positive clones were partially digested with *Sau*3AI to collect 3–6 kb DNA fragments, ligated to the *Bam*HI-linearized pBluescript II SK(+) vector, and transformed into *E. coli* DH5α cells. The transformants were grown overnight at 37 °C in LB (ampicillin100 μg mL^−1^) agar containing 0.5 % (v/v) tributyrin to assess lipolytic activity. Clones with clear halos on the screening plates were selected as positive clones and sent for further sequencing.

The nucleotide sequences of the inserted DNAs from the positive sub-clones were sequenced (BGI, Shenzhen, China). ORFs were identified using the NCBI ORF Finder (http://www.ncbi.nlm.nih.gov/gorf.html). The predicted functions of the ORFs were annotated using a BLASTP search against the NCBI non-redundant protein sequence database to determine the closest protein and microorganism (http://www.ncbi.nlm.nih.gov/blast/).

Multiple sequence alignments were carried out using the Clustal X program [45] and exported using ESPript 3.0 (http://espript.ibcp.fr/ESPript/cgi-bin/ESPript.cgi). Phylogenetic relationships among lipolytic members in each protein family were analyzed through a neighbor-joining phylogenetic analysis. One thousand bootstrap replicates were performed using the MEGA software (MEGA 6.0) [46].

### Cloning and expression of lipolytic genes in *E. coli*

Recombinant DNA techniques were performed according to standard protocols [43]. The gene *est1*, *est2*, *est3*, and *est5* generated an N-terminal His-tag of the recombinant target protein and *est4* generated a C-terminal His-tag of the recombinant target protein. All the recombinant expression plasmids were transformed into *E. coli* BL21 (DE3) or *E. coli* Top10Fʹ. The recombinant *E. coli* cells were cultivated at 37 °C in LB-Miller medium containing appropriate antibiotics. Isopropyl-β-D-1-thiogalactopyranoside (IPTG) was added (final concentration: 0.1 mM) to induce the cultures when the OD_600_ reached 0.6–0.8. The *E. coli* BL21 (DE3) and *E. coli* 10Fʹ cultures were further incubated for 20 h at 20 °C or 30 °C, respectively. The induced cells were harvested through centrifugation (5000×*g*, 10 min) at 4 °C and stored at −20 °C. The harvested cells were re-suspended in 100 mM Tris–HCl (pH 8.0) and disrupted by sonication. The cell debris was removed by centrifugation (8000×*g*, 20 min). The clear supernatants were collected and analyzed using SDS-PAGE (12.5 %) [47]. The gels were stained with Coomassie blue R250 and then destained.

### Purification of esterase EST4

The harvested cells expressing EST4 were resuspended in buffer A (50 mM Tris–HCl, pH 8.0; 300 mM NaCl; 10 mM imidazole), disrupted by sonication, and the cell lysate was centrifuged at 8000×*g* for 20 min. The resulting supernatant was loaded onto a Ni–NTA column (1 mL, Qiagen, Hilden, Germany) at a flow rate of 1.0 mL min^−1^, which was equilibrated with buffer A. The column was subsequently washed with 20 mM imidazole in buffer A to remove the impurity protein. The fractions containing the target protein were eluted with 250 mM imidazole in buffer A. The eluted protein was then combined and dialyzed extensively against Tris–HCl buffer (100 mM, pH 8.0) to remove the high concentrations of imidazole and salt [48]. Finally, the purity of the target protein was determined by SDS-PAGE.

### Enzyme characterization of esterase EST4

The characteristics of purified EST4 were determined as previously described [25]. All measurements were carried out in triplicate. The standard assays for catalytic activity were carried out using a spectrophotometric method with *p*-NP butyrate (C4) as the substrate (unless otherwise indicated) at 30 °C for 5 min. The assay mixture (1 mL) contained 1 mM *p*-NP esters and 100 mM Tris–HCl buffer (pH 8.0). After pre-incubation for 5 min, the reaction was started by addition of EST4 and terminated by addition of 1 mL of 1 % SDS. The absorbance at 405 nm was measured. One unit of esterase was defined as the amount of enzyme needed to liberate 1 μmol *p*-NP in 1 min.

The substrate range was determined under standard conditions using *p*-NP esters with acyl-chains of various lengths: *p*-NP acetate (C2), *p*-NP butyrate (C4), *p*-NP caprylate (C8), *p*-NP caprate (C10), *p*-NP laurate (C12), and *p*-NP palmitate (C16).

The optimal pH for EST4 activity was determined in the pH range from 5.2 to 10.28 under standard conditions. The following buffers were used: 100 mM sodium citrate buffer (pH 5.2–6.4), 100 mM sodium phosphate buffer (pH 6.4–8.0), 100 mM Tris–HCl buffer (pH 8.0–9.0), and 100 mM glycine-NaOH buffer (pH 9.0–10.28).

The optimal temperature for EST4 activity was measured in the temperature range from 20 to 60 °C under standard conditions. The thermostability was determined by pre-incubating the partially purified enzyme at 40–60 °C for up to 60 h and then analyzing the residual activity.

### Effect of detergents, metal ions, and organic solvents on EST4 activity

The effect of detergents on the esterase activity was analyzed by incubating the enzyme for 30 min at 30 °C in 100 mM Tris–HCl (pH 8.0) containing 0.5 % (w/v) SDS, CTAB, Triton X-100, Tween 20, Tween 40, Tween 60, or Tween 80. The effect of metal ions (CaCl_2_, MgCl_2_, MnCl_2_, ZnCl_2_, CuCl_2_, CoCl_2_, NiCl_2_, FeSO_4_, or FeCl_3_) and the chelating agent EDTA on the activity of EST4 was determined at final concentrations of 1 and 5 mM.

To estimate the organic solvent tolerance of EST4, enzyme solutions were mixed with various organic solvents at a final concentration of 20 or 50 % (v/v), as described by Li et al. [27]. The mixtures were incubated at 30 °C while shaking at 200 rpm for 12 h. The organic solvents used included dimethyl sulfoxide (DMSO), dimethylformamide (DMF), methanol, acetonitrile, ethanol, acetone, isopropanol, benzene, toluene, cyclohexane, *n*-hexane, *n*-heptane, and isooctane.

The effect of neat organic solvents on the esterase activity was investigated using the method described by Li et al. [27]. Powdered EST4 and organic solvents were mixed in sealed vessels and incubated at 30 °C while shaking at 200 rpm for 12 h. The bulk of the solvent was removed by centrifugation at 8000×*g* for 5 min, and then the residual solvent was evaporated. After the esterase was resuspended in 100 mM Tris–HCl buffer (pH 8.0), the residual activities were determined under standard conditions.

### Synthesis of short-chain flavor esters

To study the catalytic behavior of esterase EST4, short-chain flavor esters were synthesized through the transesterification of various aliphatic and aromatic alcohols with vinyl acetate as an acyl donor, under optimal reaction conditions. The cells containing esterase EST4 were centrifuged, washed once with 100 mM Tris–HCl (pH 8.0), and then lyophilized by vacuum freezing. The transesterification reactions were carried out as follows: alcohol and vinyl acetate (1:2 molar ratio of alcohol/acyl donor) were mixed with 5.0 mL of *n*-hexane, followed by the addition of 50 mg dry cell powder. The reaction mixtures were incubated in a shaking water bath at 40 °C and 200 rpm. At appropriate intervals, samples were withdrawn from the reaction mixtures and analyzed by gas chromatography (GC). A parallel reaction under the same conditions without the addition of the enzyme was used as a control. The conversion rate (%) for ester synthesis was calculated from the conversion of alcohol to ester after a given time.

### Analytical methods

The reaction samples were analyzed using a 6890 gas chromatograph (Agilent Technology, USA) equipped with a flame ionization detector (FID). The separation was performed on an HP-5 capillary column (5 % phenyl methyl siloxane capillary, 30.0 m × 250 μm × 0.25 μm nominal, Agilent Technology, USA), using *n*-octanol as an internal standard. For cinnamyl acetate, citronellyl acetate, and geranyl acetate, the column temperature was kept at 120 °C for 0.5 min, heated to 180 °C at 10 °C min^−1^, and then maintained at 180 °C for 0.5 min. For isoamyl acetate, the column temperature was kept at 90 °C for 0.5 min, heated to 130 °C at 5 °C min^−1^, and then maintained at 130 °C for 0.5 min. The injector and detector temperatures were both set to 250 °C. The conversion was calculated using the peak areas.

### Nucleotide sequence accession numbers

The nucleotide sequences of the lipolytic genes of *est1*, *est2*, *est3*, *est4*, and *est5* have been submitted to the GenBank database with accession numbers [GenBank:KT288112, GenBank:KT288113, GenBank:KT288114, GenBank:KR028985, and GenBank:KT288115, respectively].

## Additional files

10.1186/s12934-016-0435-5 Purification of metagenomic DNA by agarose gel electrophoresis. Lane 1, DNA isolated from marine mud sample; lane 2, 40-kb control DNA.
10.1186/s12934-016-0435-5 *Not*I digestion of fosmid DNA isolated from randomly chosen clones. Lines 1–10, *Not*I-digested fosmid DNA; lane 11, positive control, Fosmid cloned control DNA; lane 12, negative control, host *E. coli*; lane 13, 40-kb control insert; lane M, marker.
10.1186/s12934-016-0435-5 *Bam*HI digestion of fosmid DNA isolated from the five lipolytic clones. Lane 1, Fos-est1; lane 2, Fos-est2; lane 3, Fos-est3; lane 4, Fos-est4; lane 5, Fos-est5; lane M, 1 kb DNA Ladder.
10.1186/s12934-016-0435-5 Soluble expression of recombinant esterase EST4 in *Escherichia coli.*

10.1186/s12934-016-0435-5 Effect of various lipolytic enzymes on the hydrolysis of emulsified tributyrin. The reaction mixtures (10 mL) contained the emulsified tributyrin (50 mM) and purified proteins (20 μg) in 100 mM Tris–HCl (pH 8.0) were incubated at 30 °C for 10 min. The enzymes activity was measured by the titrimetric method and all measurements were performed in triplicate.
