# Supplementary figures and images for: A novel esterase from a marine mud metagenomic library for biocatalytic synthesis of short-chain flavor esters
Source: Microb Cell Fact. 2016 Feb 18;15:41. doi: 10.1186/s12934-016-0435-5 (PMC4758151; doi:10.1186/s12934-016-0435-5)

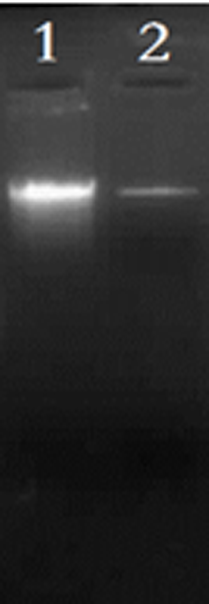

Supplement: Supplementary file 1 — 10.1186/s12934-016-0435-5 Purification of metagenomic DNA by agarose gel electrophoresis. Lane 1, DNA isolated from marine mud sample; lane 2, 40-kb control DNA. [file 12934_2016_435_MOESM1_ESM.tif]

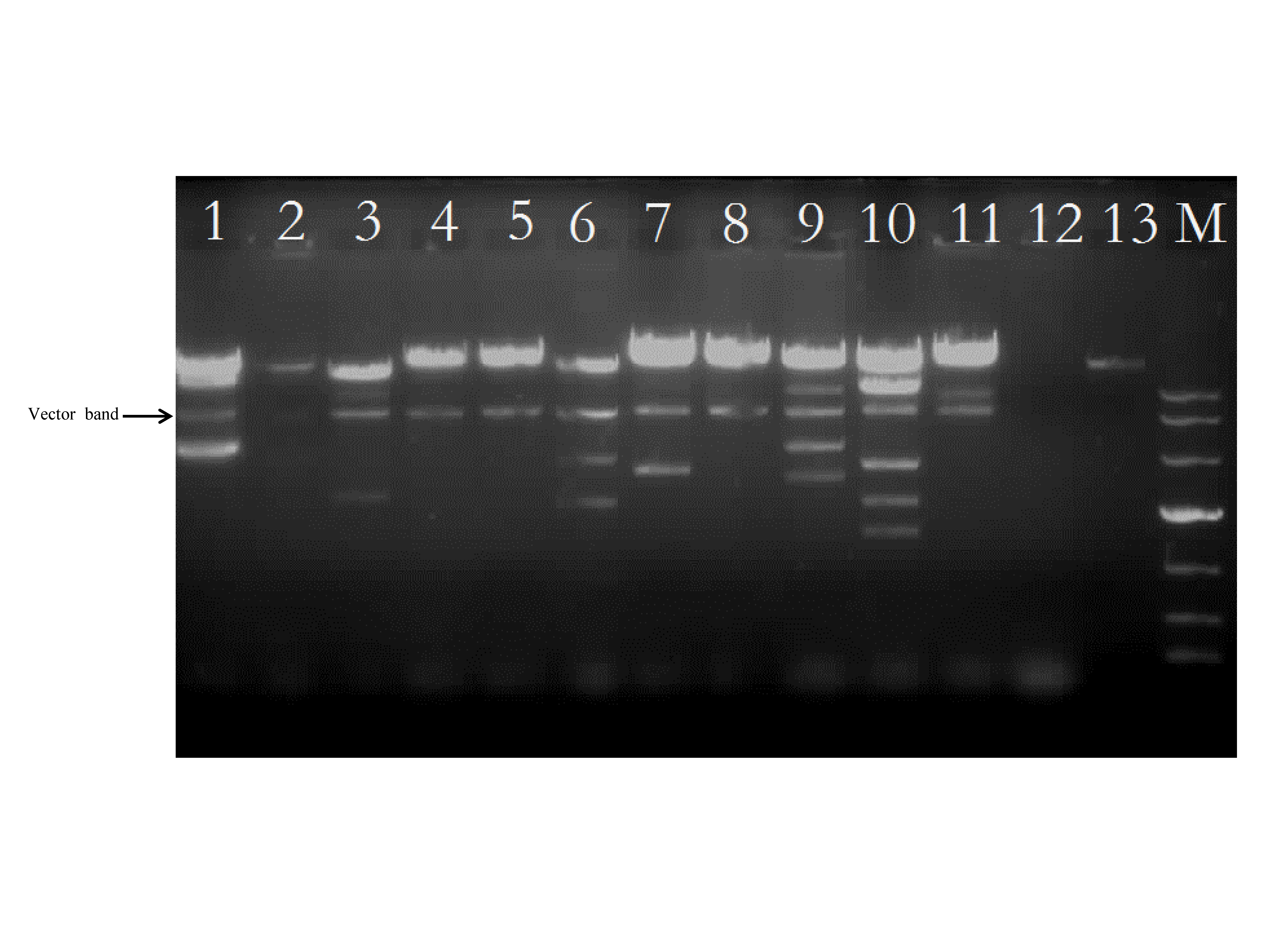

Supplement: Supplementary file 2 — 10.1186/s12934-016-0435-5 NotI digestion of fosmid DNA isolated from randomly chosen clones. Lines 1–10, NotI-digested fosmid DNA; lane 11, positive control, Fosmid cloned control DNA; lane 12, negative control, host E. coli; lane 13, 40-kb control insert; lane M, marker. [file 12934_2016_435_MOESM2_ESM.tif]

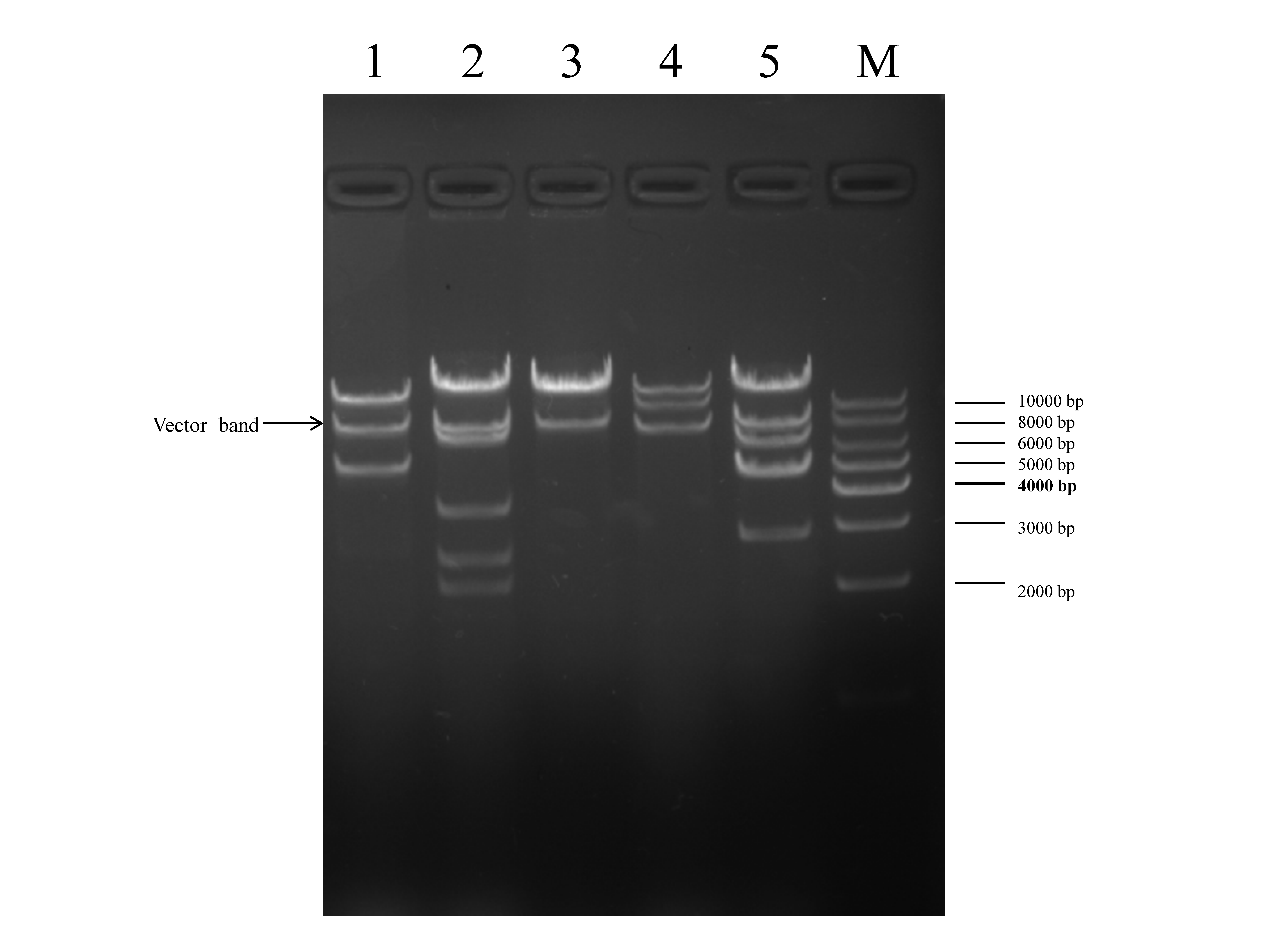

Supplement: Supplementary file 3 — 10.1186/s12934-016-0435-5 BamHI digestion of fosmid DNA isolated from the five lipolytic clones. Lane 1, Fos-est1; lane 2, Fos-est2; lane 3, Fos-est3; lane 4, Fos-est4; lane 5, Fos-est5; lane M, 1 kb DNA Ladder. [file 12934_2016_435_MOESM3_ESM.tiff]

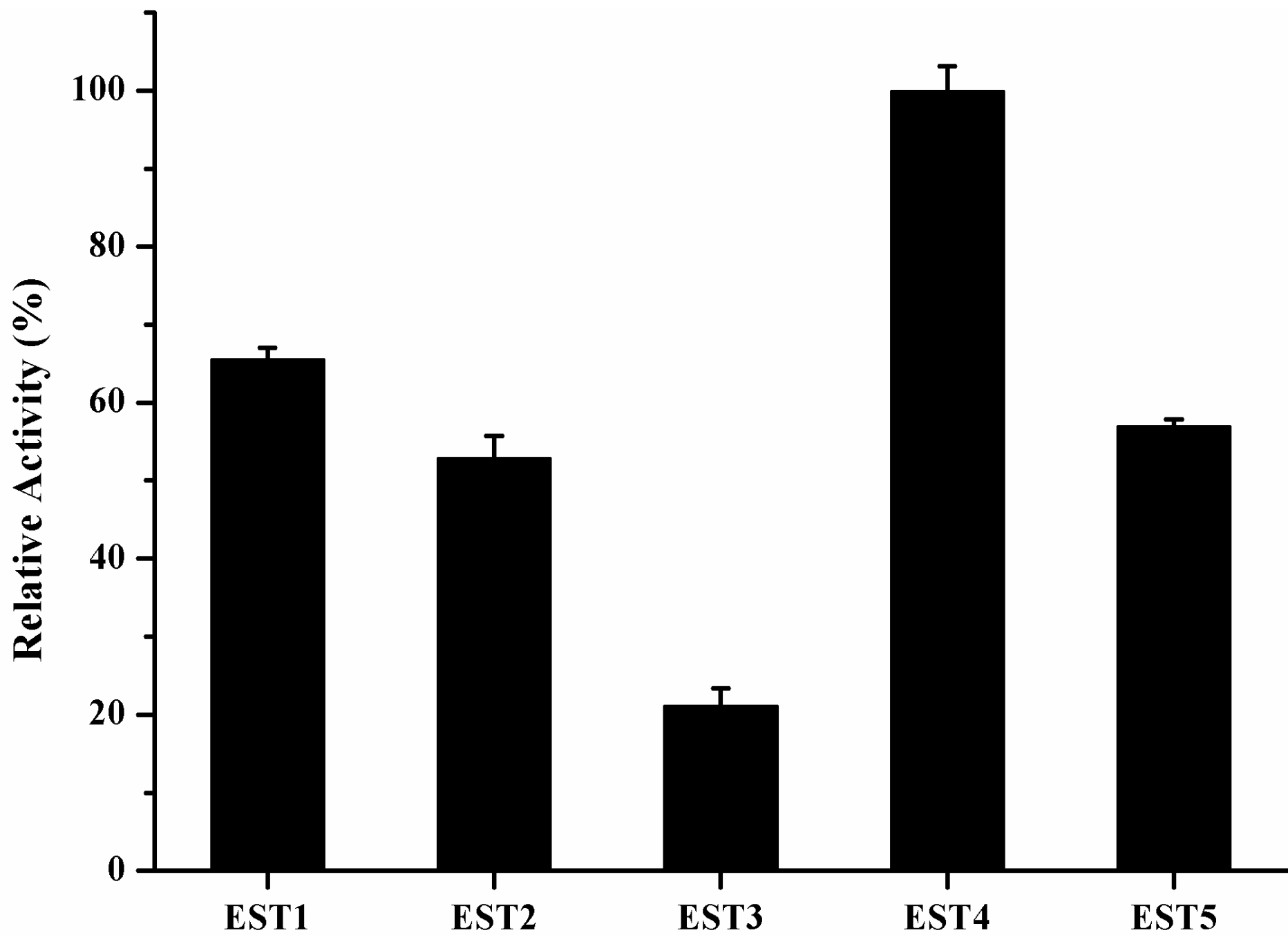

Supplement: Supplementary file 5 — 10.1186/s12934-016-0435-5 Effect of various lipolytic enzymes on the hydrolysis of emulsified tributyrin. The reaction mixtures (10 mL) contained the emulsified tributyrin (50 mM) and purified proteins (20 μg) in 100 mM Tris–HCl (pH 8.0) were incubated at 30 °C for 10 min. The enzymes activity was measured by the titrimetric method and all measurements were performed in triplicate. [file 12934_2016_435_MOESM5_ESM.pdf]
