# Supplementary material for: A novel esterase from a marine mud metagenomic library for biocatalytic synthesis of short-chain flavor esters
Source: Microb Cell Fact. 2016 Feb 18;15:41. doi: 10.1186/s12934-016-0435-5 (PMC4758151; doi:10.1186/s12934-016-0435-5)
Supplement: Supplementary file 4 — 10.1186/s12934-016-0435-5 Soluble expression of recombinant esterase EST4 in Escherichia coli. [file 12934_2016_435_MOESM4_ESM.docx]

**Additional file 4:** **[Soluble expression of recombinant esterase EST4 in](http://onlinelibrary.wiley.com/doi/10.1002/(SICI)1097-0290(19991120)65:4%3C382::AID-BIT2%3E3.0.CO;2-I/pdf) *[Escherichia coli](http://onlinelibrary.wiley.com/doi/10.1002/(SICI)1097-0290(19991120)65:4%3C382::AID-BIT2%3E3.0.CO;2-I/pdf)***

The gene *est4* was first expressed in *E. coli* BL21 (DE3)/pET-28a (+) with an N-terminal His_6_-tag, however, it was expressed in the form of inclusion body even after the optimization of culture temperatures (20 °C, 30 °C, and 37 °C) and the concentrations of IPTG. In order to obtain a sufficient amount of the esterase EST4 in an active form, combinations of a series of hosts and expression vectors (*E. coli* BL21 (DE3)/pET-21a (+), *E. coli* BL21 (DE3)/pET-32a (+), *E. coli* BL21 (DE3)/pET-42a (+), *E. coli* M15/pQE-30, and *E. coli* Top10Fʹ/pLLP-OmpA) were tested by considering the impacts of the strength of the promoter, different fusion tags, and signal peptide on the soluble expression of protein. To avoid the influence of fused His_6_-tag to the expression of protein, the *est4* gene was expressed in pET-21a (+) as single protein which had no fused tag compared with that it was in pET-28a (+). Based on the possibility that some soluble tags could enhance the solubility of expressed proteins in the form of fused protein, the gene *est4* was fused with the 109 amino acids Trx-tag in the pET-32a (+) and the 220 amino acids GST-tag in the pET-42a (+). In terms of promoter, the gene *est4* was placed under the control of the weaker T5 promoter in pQE-30 vector compared with the strong T7 promoter in pET-28a (+). Unfortunately, all the efforts failed to obtain the soluble expression of target protein. Different from the other vectors which express protein into the cytoplasm, the pLLP-OmpA vector can secrete the targeted protein to the periplasmic space through an *omp*A signal. Finally, the gene *est4* was successfully overexpressed in an active form in *E. coli* Top10Fʹ/pLLP-OmpA with a C-terminal His_6_-tag (Figure 3). The soluble expression of esterase EST4 in *E. coli* Top10Fʹ may be due to the oxidizing environment of the periplasm that facilitates proper protein folding.
